# Reliable genomic strategies for species classification of plant genetic resources

**DOI:** 10.1186/s12859-021-04018-6

**Published:** 2021-03-31

**Authors:** Artur van Bemmelen van der Plaat, Rob van Treuren, Theo J. L. van Hintum

**Affiliations:** Centre for Genetic Resources, Wageningen University and Research, P.O. Box 16, 6700 AA Wageningen, The Netherlands

**Keywords:** Plant genetic resources, Species classification, Machine learning, Crop wild relatives, Gene bank documentation, Genomics

## Abstract

**Background:**

To address the need for easy and reliable species classification in plant genetic resources collections, we assessed the potential of five classifiers (Random Forest, Neighbour-Joining, 1-Nearest Neighbour, a conservative variety of 3-Nearest Neighbours and Naive Bayes) We investigated the effects of the number of accessions per species and misclassification rate on classification success, and validated theirs generic value results with three complete datasets.

**Results:**

We found the conservative variety of 3-Nearest Neighbours to be the most reliable classifier when varying species representation and misclassification rate. Through the analysis of the three complete datasets, this finding showed generic value. Additionally, we present various options for marker selection for classification taks such as these.

**Conclusions:**

Large-scale genomic data are increasingly being produced for genetic resources collections. These data are useful to address species classification issues regarding crop wild relatives, and improve genebank documentation. Implementation of a classification method that can improve the quality of bad datasets without gold standard training data is considered an innovative and efficient method to improve gene bank documentation.

**Supplementary information:**

The online version contains supplementary material available at 10.1186/s12859-021-04018-6.

## Background

The goal of gene banks is to secure genetic resources for research and breeding now and in the future. In 2009, gene banks worldwide maintained an estimated 7.4 million accessions, 1.4 million more than in 1996 [1]. Roughly 30% of this increase is accounted for by the increased interest in crop wild relatives (CWR), which include the progenitors of domesticated crops as well as species closely related to them. The use of crop wild relatives to improve crop yield, pest and disease resistance, and tolerance for biotic and abiotic stress is well established, with important examples dating back more than 60 years [2]. Since the introduction of marker assisted breeding and more advanced technologies, the use of crop wild relatives has only intensified [3].

The increased interest in a broad range of crop wild relatives also necessitates expertise in species identification, as the distribution of misidentified plant materials can have significant adverse effects on the subsequent use. Traditionally, species identification has been the domain of taxonomists, who identify species based on morphological features. This is a time-consuming task, while limited morphological variation may still cause unreliable identifications [4, 5]. In addition to initial misclassifications, gene bank documentation may contain errors due to complicated accession histories involving exchanges among institutions and multiple rounds of regeneration. As a result, mistaken identities in genetic resources collections are not uncommon. Therefore, efficient methods to identify and correct species misclassifications would be very helpful to gene banks.

The need for easy and reliable species identification is not restricted to gene banks. It has existed for much longer, in disciplines ranging from ecology to food fraud detection [6], and gave rise to the conception of DNA barcoding in 2003. DNA barcoding is a taxonomic method that uses variation in the mitochondrial gene cytochrome *c* oxidase I (*cox1*) for species identification [7]. Since the first publication, DNA barcoding has received wide support for its straightforward approach and efficacy in both the identification of biological specimens and the discovery of species [8, 9]. However, some criticisms have been levelled at the method as well, directed at its departure from classic taxonomy by using genetic distance measures instead of character based identification, the lack of an objective set of criteria to delineate species when using these distance measures, and whether using only the *cox1* gene is really sufficient [10].

Although *cox1* has been shown to be successful in identifying species of butterflies, birds, bats, fish, and mosquito [11–15], *cox1* shows insufficient variation to distinguish species in various other groups, such as vascular plants, fungi, invertebrates, reptiles and amphibians [16–19]. An alternative to *cox1* in these groups remains elusive, but it is evident that the species resolution of DNA barcoding benefits from including additional loci in the analyses to increase the number of divergent sites [20, 21].

Still, the literature addressing the methodological shortcomings of DNA barcoding is valuable and very informative. The classification performance of many candidate methods has already been analyzed and compared in DNA barcoding, such as such as Neighbour-Joining (NJ), k-Nearest Neighbours (k-NN), Classification and Regression Trees (CART), Random Forest, kernel methods, Naive Bayes classifiers, Repeated Incremental Pruning to Produce Error Reduction (RIPPER) [22], Support Vector Machines (SVMs), BLOG [23], and DNA-BAR [24–28]. The overlap of candidate methods between studies, however, is sparse. We selected a variety of methods from a pool of successful candidate methods, and aimed for diversity in methodology. This resulted in the selection of Random Forest, NJ, k-NN (at k = 1 and k = 3), and Naive Bayes. The first three were the most promising methods in the comparison study of Austerlitz et al*.* [26], whereas Naive Bayes was one of the best performers in the study of Weitschek et al*.* [27]. These methods broadly constitute three types of approaches: distance methods (k-NN), phylogenetic methods (NJ), and supervised machine learners (Random Forest, Naive Bayes). In these comparisons, NJ will be representative of the commonly used methodology to correct misclassifications in diverse datasets, as these are currently based on phylogenetic analysis.

In this paper, we depart from the single gene approach of DNA barcoding strategies and instead employ SNPs from throughout the genome. This will benefit gene banks in three ways. Firstly, methods will be more generalizable across species as there will be more variation to utilize in species delineation. Secondly, the methods will be applicable to a broader range of genotyping datasets, including non-sequencing methods such as AFLPs. Thirdly, in their criticism of DNA barcoding, many have pointed out that any method relying on a single gene will encounter a problem in detecting and classifying hybrid introgressions [29–31], information which will be of interest for germplasm end-users. Although our datasets don’t include enough confirmed hybrid accessions, we expect that genome-wide approaches will be more successful in identifying the major donor species of a hybrid.

For the vast majority of crop wild relatives, a verified genomic dataset with which to train classification models is lacking. For a select number of crops, the creation of such a dataset will only be a matter of time, but for most crops the economic incentive is lacking. In the short- and long-term, the genetic resources community would therefore benefit from a classification strategy that does not require a perfectly classified training set, but will instead work with datasets as they are available for genetic resources collections, i.e. mostly verified but misclassifications may be present. If the development of such a strategy is successful, the genomic data that are already available can immediately be used to improve the classification accuracy of the collection.

There are a number of difficulties to this development. Firstly, there are multiple dataset characteristics that have been shown to impact classification success in DNA barcoding [26], such as the number of species, their respective speciation time, and the number of accessions per species. These characteristics will likely also affect our classification models. Supervised learners in particular (e.g. Random Forest and Naïve Bayes) may need more accessions per species to perform well. To test at what point, if any, machine learners are no longer recommended, curated datasets are created to study the effect of the number of accessions per species on the performance of classifiers.

Secondly, classification models should be able to learn from bad training data, i.e. training data with misclassifications. To determine which classifiers (if any) are most suited to work with imperfectly classified data, we simulated different misclassification rates. Comparison between the applied misclassification rate and the classification success of classifiers should reveal whether the classifiers succeeded in improving the quality of the dataset.

Thirdly, a rather severe imbalance in species representation is found in CWR datasets. Wild relatives that will readily exchange genes of interest with their cultivated counterparts (species belonging to the primary gene pool) are much higher represented in datasets than wild relatives from the secondary and tertiary gene pool, as these datasets are usually generated for breeding purposes. To determine how well our results translate to such datasets, we tested the classifiers on three complete datasets, and used cross-validation on the supervised machine learners to how much of their initial success may be due to over-fitting.

The goal of this work is to lay the basis for curators of genetic resources to discover possible misclassifications in genotyped collections, regardless of species, inclusion of wild relatives, or genotyping method. This will improve the quality of collections at minimal cost, and contribute towards making bioinformatics more accessible to genetic resource specialists.

## Results

### Performance on curated datasets

To determine if classifiers can improve the quality of a bad training dataset, they were trained on curated *Helianthus* datasets with varying rates of artificially induced misclassifications. They then classified these curated datasets. To examine the impact of species representation on this process, the number of representatives per species was also varied. Through 5,000 repetitions of artificially induced misclassifications in different curated datasets, the best classifiers for each of these datasets were identified (Table 1).

**Table 1:** Median prediction accuracy in the 15 × 5,000 curated *Helianthus* datasets

Accessions per species	Misclassification rate (%)	RF	NB	NJ	1-NN	3-NN
	6.25	0.63	**0.88**	0.81	0.81	0.75
2	12.50	0.56	**0.75**	**0.75**	**0.75**	0.63
	18.75	0.50	**0.69**	**0.69**	**0.69**	0.63
	6.25	0.84	**0.97**	0.91	0.78	**0.97**
4	12.50	0.81	0.88	0.84	0.75	**0.94**
	18.75	0.78	0.81	0.78	0.69	**0.88**
	6.25	**0.96**	**0.96**	**0.96**	0.94	**0.96**
6	12.50	**0.94**	0.85	0.92	0.88	**0.94**
	18.75	**0.90**	0.77	0.88	0.81	0.88
	6.25	0.95	0.88	0.94	0.83	**0.98**
8	12.50	0.94	0.81	0.89	0.78	**0.95**
	18.75	**0.92**	0.70	0.84	0.72	0.91
	6.25	**0.98**	0.88	0.93	0.91	**0.98**
10	12.50	**0.96**	0.75	0.89	0.85	0.94
	18.75	**0.96**	0.65	0.85	0.79	0.89
	Median	0.92	0.81	0.88	0.79	0.94

Classifiers are Random Forest (RF), Naive Bayes (NB), Neighbour-Joining (NJ), 1-Nearest Neighbour (1-NN), and 3-Nearest Neighbours (3-NN) respectively. For each parameter combination, the highest median score is presented in bold

When the species representation exceeded 4, Random Forest was the best classifier. When species representation was lower, Naive Bayes performed markedly better than Random Forest. Overall, 3-NN showed the best performance (median prediction accuracy of 0.94 vs Random Forest’s 0.92). Random Forest and 3-NN have proven themselves adept at improving the quality of a bad dataset, and to provide a significant improvement over NJ, the method that represents the current methodology to address misclassifications.

Regardless of the quality of the curated datasets, NJ was outperformed. With more optimal datasets, specifically datasets including 10 accessions per species and a misclassification rate of 6.25%, NJ struggled to improve the quality. Random Forest and 3-NN, by comparison, reduced the misclassification rate in these datasets to a median of 2%. With less optimal datasets, in this case 4 accessions per species and a misclassification rate of 12.50%, 3-NN reduced the misclassification rate to a median of 6%, a marked improvement. In contrast, NJ actually increased the misclassification rate of these datasets and output data with a median misclassification rate of 16%.

In all cases, the classifiers showed reduced performance as misclassification rate rose. Yet surprisingly, the misclassification rate appears to have little influence on the best classifier. Exceptions were observed for datasets with 4 or 8 accessions per species, but the difference in prediction accuracy was only minimal in these cases. It is possible that this effect (or lack thereof) is caused by the procedure used to induce misclassifications. Because accessions to misclassify were selected just as randomly as the species to mutate their identity to, all species were affected by these artificial misclassifications at similar rates. This random misclassification effect should be much easier for classifiers to mitigate than the more structural nature of misclassifications one would expect when two or more morphologically similar species are systematically confused.

### Performance on complete datasets

To test whether the conclusions of the curated datasets would hold and would show generic value, we compared the performance of the classifiers on three unmodified complete datasets. For this purpose, we acquired an unbiased estimate of prediction accuracy of the supervised machine learners we acquired an unbiased prediction estimate through leave-one-out cross-validation or bagging. The distance-based methods classified the data as before. Additionally, classification performance was quantified by prediction accuracy per species [see Additional file 1]. These tables show 3-NN as the best performing classifier. The performance of 3-NN is consistent with the results of the curated datasets. As expected based on the results of the curated datasets, the performance of Random Forest improved when species were represented by more accessions. The overall difference between RF, NJ, and 1-NN, however, appears slight.

Perhaps most surprising result is the extreme poor performance of Naive Bayes. It performed best in the resequenced tomato dataset, in which its correct classifications consist almost exclusively of the species with the largest representation, *S. lycopersicum* and *S. habrochaites*. Conversely, it misclassified every single one of the 100 *H. annuus* accessions, which suggests that species representation is not solely at the root of the poor performance.

As expected based on the curated datasets, Random Forest performed best on the AFLP tomato dataset, which contained the fewest species represented by 4 or less accessions. There was no difference between the out-of-bag prediction accuracy, and the fraction of accessions that was correctly classified. This is unsuprising with forests with 10,000 trees in with relatively small datasets.

## Discussion

The aim of this research was to identify the most reliable methods for genome-wide species classification of imperfectly classified datasets. We used methods that previously proved successful in DNA barcoding and invesigated their performance under varying rates of misclassification and species representation on genome-wide SNPs We then assessed their performance on three complete datasets. Here we reflect on the methodology used in this research, as well as specify the methodologies we recommend to the genetic resources community.

### Simulated misclassifications

To determine the effect of misclassification rate on classifier performance, misclassifications were simulated by randomly changing an accessions’ species to a random different species from the same dataset. This resulted in a reduced performance for all classifiers as the misclassification rate rose, yet surprisingly, the misclassification rate appeared to have little influence on the best classifier. Exceptions were observed for datasets with 4 or 8 accessions per species, but the difference in prediction accuracy was only minimal in these cases. It is possible that this effect (or lack thereof) is caused by the method used to induce misclassifications. Accessions to misclassify were selected randomly, and as such, all species were affected by these artificial misclassifications at similar rates. This effect might be much easier for classifiers to mitigate than the more structural nature of misclassifications one would expect when two or more morphologically similar species are systematically confused.

### Validity of outlier detection methods

For the curation of the *Helianthus* datasets, potential misclassifications in a subset of sunflower species were identified based on either their outlying position in the neighbour-joining tree [Additional file 2], or their relatively small proximity to others of their class in a Random Forest [see Additional file 3]. We reexamined these potential misclassifications using the complete sunflower dataset. For this, we compared a priori classifications, and predictions of both Random Forest and 3-NN, the most reliable classification methods.

The performance of the Random Forest outlier detection method was unexpectedly poor, as only two out of six (*max148* and *niv07*) accessions marked as outliers were actually re-classified by Random Forest and 3-NN. Comparison of suspected outliers with non-outliers revealed that considerably fewer reads were generated for outliers (median 1.0 million vs 2.4 million). This strongly suggests Random Forest used the number of imputed values to distinguish outliers from non-outliers. We used the most common allele at each locus to impute missing values, which in this case is likely the allele belonging to *Helianthus annuus*, which is represented by the vast majority of the accessions (Table 2). This way, we likely introduced *Helianthus annuus* alleles in accessions that were not *Helianthus annuus*, which led to their relative dissimilarity to others of their species. Interestingly, Random Forest was robust enough to confirm the a priori classifications despite this unfortunate artefact of the imputation method. This finding shows both the robustness of Random Forest classification, but also the sensitivity of the Random Forest outlier detection technique. Still, we do not recommend using Random Forest outlier detection technique for datasets with missing values imputed using the most common allele at each unknown locus, because the combination seems especially prone to false positives.

The performance of NJ-based outlier detection fared much better. All accessions marked as outlying, with the exception of *pet02*, were found to be a different species by Random Forest and 3-NN classification. At first glance [see Additional file 1] *pet02* seems distant from the cluster of other *Helianthus petiolaris*, but rotation of subtrees could position it much closer. How close is close enough to not be considered an outlier? This is a technique that uses human judgment, and this accession shows that interpreting phylogeny through trees can be rather tricky. Instead of this technique, we recommend using classification methods as outlined in the section "Practical Recommendations".

### Challenges in species classification

Some of the species represented in the datasets are notably harder to classify than others with similar species representation, tomato species *S. corneliomulleri* and *S. peruvianum* senso stricto in particular. Nearly all classification mistakes involving these species, mixed up the two (Table 3). These are two of four species into which *S. peruvianum* sensu lato was recently split [51, 52]. Peralta et al*.* describe their approach towards this delineation as combining morphological, molecular, and ecological data, as well as having relied on clear morphological discontinuities to define entities. However, none of the strict consensus trees, based on either GBSSI gene sequences, AFLP data, or morphological characters presented by Peralta et al*.* [52] show delineation between these two species. This finding has since then been reproduced several times [43, 53, 54]. Moreover, no significant difference between the environments *S. corneliomulleri* and *S. peruvianum* s.s. inhabit was found either [55]. This lack of delineation clearly affected distance-based methods 1-NN and NJ, whereas 3-NN appears a bit more succesful. Peralta et al*.* cite incomplete lineage sorting as explanation, a characteristic which would indeed foil distance-based methods such as phylogenetic trees, but should have left a supervised machine learner like RF mostly unaffected. Random Forest, however, was not able to distinguish these species any better than 3-NN.

Conversely though similarly, a recent study on gene flow between sunflower species *H. petiolaris* and *H. neglectus* found it was unlikely that these two populations represent two distinct isolated gene pools [56]. The authors argued therefore that the populations currently recognized as *H. neglectus*, do not warrant recognition as a distinct species but should instead be recognized as a subspecies of *H. petiolaris*. Despite this finding, RF, NJ, and 3-NN distinguished *H. petiolaris* and *H. neglectus* with success (Table 2).

**Table 2:** Confusion matrix of *H. petiolaris* and *H. neglectus* in the sunflower dataset

	RF			NB			NJ		
A priori classification	*H. pet*	*H. neg*	Other	*H. pet*	*H. neg*	Other	*H. pet*	*H. neg*	Other
*H. petiolaris* (n = 18)	1.00	0.00	0.00	0.00	0.00	1.00	0.94	0.06	0.00
*H. neglectus* (n = 19)	0.11	0.84	0.05	0.00	0.00	1.00	0.05	0.84	0.11

	1-NN			3-NN		
A priori classification	*H. pet*	*H. neg*	Other	*H. pet*	*H. neg*	Other
*H. petiolaris* (n = 18)	0.11	0.00	0.89	1.00	0.00	0.00
*H. neglectus* (n = 19)	0.00	0.53	0.47	0.05	0.89	0.05

Confusion matrix showing fractionally how often *H. petiolaris* and *H. neglectus* are classified as themselves, as each other, and as other species by Random Forest (RF), Naive Bayes (NB), Neighbour-Joining (NJ), 1-Nearest Neighbour (1-NN), and 3-Nearest Neighbours (3-NN)

While the sample sizes of this experiment are insufficient to draw conclusions, these findings suggest it might be fruitful to use classification methods alongside statistical methods when testing whether populations possess distinctive qualities.

### The variable success of Naive Bayes

When comparing Table 4 with Table 3, it is evident that the prediction success of Naive Bayes is highly variable. Comparison of its performance on *Solanum lycopersicum* (Additional file 4) and *Helianthus annuus* (Additional file 5) suggests that this variability is not solely due to species representation. Rish et al*.* (2001) show that Naive Bayes reaches its best performance in two opposite cases: completely independent features and functionally highly dependent features [57]. These cases might translate to these optimal cases: classification of a trait unrelated to lineage (completely independent), or classification in species with very low intraspecific diversity (highly dependent). This hypothesis would be consistent with a good classification performance on *S. lycopersicum*, as the accessions that represent it are all cultivated material and have very low diversity, and a bad performance on *Helianthus annuus*, the progenitor of cultivated sunflower, which has one of the highest rates of genetic diversity among wild sunflowers [58].

**Table 3:** Confusion matrix of *S. peruvianum* and *S. corneliomulleri* in the AFLP dataset

	RF			NB			NJ		
A priori classification	*S. per*	*S. cor*	Other	*S. per*	*S. cor*	Other	*S. per*	*S. cor*	Other
*S. peruvianum* (n = 12)	0.92	0.08	0.00	0.00	0.00	1.00	0.75	0.25	0.00
*S. corneliomulleri* (n = 4)	1.00	0.00	0.00	0.00	0.25	0.75	0.50	0.50	0.00

	1-NN			3-NN		
A priori classification	*S. per*	*S. cor*	Other	*S. per*	*S. cor*	Other
*S. peruvianum* (n = 12)	0.75	0.25	0.00	1.00	0.00	0.00
*S. corneliomulleri* (n = 4)	0.75	0.25	0.00	0.25	0.67	0.25

Confusion matrix of the AFLP tomato dataset, showing fractionally how often *S. peruvianum* and *S. corneliomulleri* are classified as themselves, as each other, and as other species by Random Forest (RF), Naive Bayes (NB), Neighbour-Joining (NJ), 1-Nearest Neighbour (1-NN), and 3-Nearest Neighbours (3-NN)

**Table 4:** Prediction accuracy per complete dataset.

	RF OOB	NB LOO	NJ	1-NN	3-NN
Resequenced sunflower	0.86	0.07	0.91	0.81	**0.96**
AFLP tomato	0.92	0.14	0.84	0.86	**0.95**
Resequenced tomato	0.85	0.75	0.85	0.88	**0.94**

The supervised machine learners (RF and NB) have been crossvalidated as described in the Methods section. The best performance for each dataset is presented in bold

### Options for marker selection

There are no definite guidelines on how best to select markers from resequenced data sets and reduce them to a computationally more manageable number. We briefly tested two different strategies, namely (1) applying a strict filter to select only what one would perceive as high quality markers, and (2) randomly thinning the markers to a desired number. In the resequenced tomato data set, we found that filtering the markers (as opposed to thinning) led to a great decrease in classifier performance (median prediction accuracy across classifiers of 0.91 vs. 0.73). In the resequenced sunflower dataset we found that the effect was opposite (0.82 vs. 0.90). By testing both strategies and choosing the marker selection with the best results, we were able to achieve good prediction accuracy for all complete datasets. We therefore believe these strategies to be sufficiently sound for use in species classification. These strategies can be implemented using command line variant filtering tools such as VCFTools or Plink (which are very fast but currently only available on Linux or MacOS), or on Windows machines using R [59, 60] or Python[61].

Additionally, other options exist for marker selection, including using only variant sites present in orthologous genes [41], variant-pruning based on linkage disequilibrium [58], or even reference free comparisons [62–65]. Reference free strategies are expected to be less successful as genome coverage drops and will require the raw sequence reads (fastq files) instead of variant call files, but may otherwise be very effective in species lacking a reference genome. Among reference free methods, DiscoSNP++  [65] in particular prides itself on its user-friendliness, as it needs relatively little RAM memory and computational time, and could therefore be run on a desktop computer. Overall, the choice for any particular method may be constrained by user expertise, computational capacity, sequencing depth and quality, and the availability of a suitable reference genome.

## Conclusions

Gene banks play a crucial role in securing genetic diversity for research and breeding, now and in the future. The collection and correct classification of crop wild relatives is an important aspect of this work. Classifying accessions based on morphological features alone, however, is time-consuming and error prone. As collections of crop wild relatives are increasingly genotyped and sequenced, this creates an excellent opportunity for gene banks to improve the quality of their documentation by identifying and correcting misclassifications. Gold standard datasets, however, are lacking for many crops and crop wild relatives. As such, the ambitious premise of this work was to find the best method for species classification, regardless of species, inclusion of wild relatives, or genotyping method, while working with imperfectly classified datasets.

We found that a conservative variety of 3-Nearest Neighbours is particularly suited to improve the quality of a bad dataset, and is a significant improvement over Neighbour-Joining, which represents the current phylogenetic methodology to address misclassifications. Based on its performance on the three complete datasets, we feel confident that this variety of 3-Nearest Neighbours will reliably perform well on a large variety of datasets.

There are still more avenues to explore regarding the use and improvement of bad training data in species classification tasks, but based on this research, we have formulated practical recommendations that can be used immediately by curators of genetic resources collections.

Furthermore, based on these findings and recommendations, a simple software tool could be developed to assist plant genetic resources curators in identifying potential misclassifications, using the current classifications and genomic data. Such a tool could eventually be developed further to study other descriptors, such as disease susceptibility, and to predict the likelihood of accessions being resistant and the likelihood of the prediction being correct. This has the potential to increase the quality of gene bank documentation tremendously, and thus increase the value of these priceless plant genetic resources.

## Methods

To identify the flaws of various classification methods, we used curated but highly diverse datasets of sunflower. We artificially varied species representation (number of accessions per species) and misclassification rate (fraction of misclassified accessions) in these data sets, and used five different classification methods to correct the misclassifications introduced. We then verified the generic value of these methods by applying them to three complete datasets.

### Classification methods

We selected classification methods based on their success in DNA barcoding studies, and aimed for diversity in methodology. This resulted in the selection of Random Forest, NJ, k-NN, and Naive Bayes. The first three were the most promising methods in the comparison study of Austerlitz et al*.* [26], whereas Naive Bayes was one of the best performers in the study of Weitschek et al*.* [27]. These methods broadly constitute three types of approaches: distance methods (k-NN), phylogenetic methods (NJ), and supervised machine learners (Random Forest, Naive Bayes). In these comparisons, NJ will be representative of the commonly used methodology to correct misclassifications in diverse datasets, as these are currently based on phylogenetic analysis.

As genebanks often work with species for which there are currently no gold standard classified datasets, the aim of this research is to find classification methods that can learn from bad training data, in such a way that they can improve the quality of the same data by reducing the number of misclassifications. We use a curated dataset with artificially introduced misclassifications to verify if models can actually improve the quality of the data, or if the models will output the same or even worse quality data when working with a bad training dataset.

### Random forest

Random Forest is an algorithm that combines hundreds or thousands of decision trees, trains each one on a slightly different set of observations through bootstrapping, and splits each decision node based on a random subset of features (e.g. molecular markers). The forest will classify new samples by funneling them down all decision trees, and adopting the classification proposed by the majority of the trees [32]. This averaging of predictions (called bagging, or bootstrap aggregating), combined with the bootstrapping of the observations improves the stability and accuracy of predictions, and helps to avoid over-fitting.

To implement the Random Forest algorithm, R package *ranger* [33] was used. This package is true to the original algorithm, but boosts computational efficiency through parallel processing. *ranger* was run with replacement with 10,000 trees, the default *mtry* value of ^*√*^*p*, and the gini impurity split rule. Samples were classified by *ranger* internally, by only using the trees each sample was out of bag for. This means that each sample was effectively classified by 0.368 × 10,000 = 3,680 trees, hence the high number of trees initially chosen.

Because Random Forest does not allow for any missing data, values were imputed with the *na.roughfix* function from R package RandomForest [34]. This method replaces the missing allele at each site with the most common one. Although this imputation method is not very sophisticated, it is very fast, makes no assumptions about the data, and works independently of any class information.

### Neighbour-joining

Neighbour-Joining (NJ) is a phylogenetic clustering method that constructs a tree from a distance matrix [35]. This method was implemented using the functions *dist.gene* and *nj* from R package APE [36], and additionally a script to classify the samples based on the constructed NJ tree. This script was based on the description of Austerlitz et al*.*, in their paper comparing various classification methods for DNA barcode analysis [26]. The distance matrix was computed with *dist.gene* with pairwise deletion enabled. With this option, *dist.gene* constructs a distance matrix by determining the number of divergent sites through pairwise comparison, and discarding the markers for which data of one or both samples is missing. The classification script reads the NJ tree and assigns the query sample the majority species of the smallest subtree it occurs in. If no majority is found, the process is repeated with the second-to-one smallest subtree the query occurs in. If no majority species emerges in this subtree either, the query is determined to be ambiguous.

#### k-nearest neighbours classification

We used two different Nearest Neighbours strategies, which vary in k number and distance measure. The first strategy is 1-Nearest Neighbour (1-NN), which assigns the query sample to the species of the most similar sample within the examined dataset. This strategy causes a problem when two nearest neighbours don’t share the same identity. Take, for example, a case of 2 neighbouring samples that are the same species, one a priori classification may be correct, and the other incorrect. 1-NN will assign the correct identity to the misclassified sample, but then go on and assign the incorrect identity to the other sample.

Nevertheless, we included 1-NN to put the results of other classification methods into perspective, because we consider a method that cannot outperform 1-NN unsuitable for implementation. To our knowledge, there is no R package that offers nearest neighbour classification with built-in leave-one-out cross-validation, so a custom function was written that computes the distance matrix only once, and then classifies the accessions while ignoring each query sample’s a priori classification. Distance between samples was determined by APE’s *dist.gene* function with pairwise deletion enabled. *dist.gene* performs a pairwise comparison for all samples and presents a conservative estimate of the number of divergent sites by ignoring all sites with missing values for one or both samples. The most similar sample is then selected and its species identity is assigned to the query sample.

The second strategy is a conservative variety of 3-Nearest Neighbours (3-NN), which includes the query sample itself among the three selected neighbours. The inclusion of the query sample amoung the neighbours increases the burden of evidence to overturn the a priori classification, as only one neighbour is needed to confirm it, while two are needed to overturn it in a majority vote. Simultaneously, this decreases the bare minimum of accessions a species needs for unambiguous classification from 3 to 2. If there are ties for the third nearest sample, all candidates are included in the vote. If no majority is reached, the sample is classified as ambiguous. 3-NN was implemented using the *knn* function from R package class, using *k* = *3, l* = *2*, and *use.all* = *TRUE*. This function uses Euclidian distance to determine similarity instead of the number of divergent sites, as used for 1-NN and NJ. Because this function does not allow missing data, missing data were imputed in the same manner as for Random Forest.

### Naive bayes classifier

The Naive Bayes classifier is a probabilistic classifier based on Bayes’ theorem. Bayes’ theorem describes the probability of example *E* being a member of class *C*, based on prior knowledge of prediction features that might be related to this class. For example, if cancer is related to age, then with Bayes’ theorem, a person’s age can be used to more accurately assess the likelihood of them having cancer, compared to assessing the probability of cancer without this knowledge. The "naive" aspect of this classifier results from its assumption that each predictor is independent from all others. In our study, this amounts to disregarding linkage between markers. The independence assumption of Naive Bayes is rarely warranted, but works surprisingly well in practice. Zhang (2004) explored potential causes of this paradox and showed that Naive Bayes does not need true independence of predictors to perform optimally, but rather demands an even distribution of dependencies in classes, or dependencies that cancel each other out [37]. In DNA barcode classification, Naive Bayes has been found successful [25]. The authors claim that ignoring dependence between predictors can lead to poorly estimated class probabilities, but will still result in correct classifications if the correct group is the most probable. The Naive Bayes model was created with the *naiveBayes* function from R package e1071 [38], using a value of 1 for Laplace smoothing. Predictions were made using the *predict* function from e1071. The Naive Bayes classifier does not require missing data to be absent, but performs much better with a well-imputed dataset. Missing data were therefore handled in the same manner as for Random Forest, i.e. by imputing the most common allele at each site.

### Selected datasets

The datasets selected for the evaluation of classification methods were chosen based on their high number of crop wild relatives included, as well as their respective differences in species representation. The characteristics of the datasets are summarized in Table 5.

**Table 5:** Characteristics of the datasets used to compare the classifiers.

Crop	Type	Accessions	Species	Markers	Reference
Sunflower	Resequenced	287	21	15,285	Baute et al. [39]
Tomato	AFLP	210	16	219	Zuriaga et al. [43]
Tomato	Resequenced	80	13	100,000	100 Tomato Genome Sequencing Consortium et al. [41]

In dataset of the 100 Tomato Genome Sequencing Consortium et al., we only included accessions of unadmixed ancestry. The number of markers listed is the number remaining after filtering

The dataset chosen to create the curated datasets is a resequenced sunflower dataset [39], which includes 22 wild sunflower species. Of these species, 8 are represented by 10 or more accessions. The median number of accessions per species is 6.5. To simplify analyses and boost computation speed, the variant sites were filtered for > 80% call rate, > 1% minor allele frequency, no indels, and a minimum of 200,000 reads per accession using VCFTools version 0.1.15 [40]. Additionally, individual genotypes were filtered to remove calls with < 5 reads. After filtering, 15,285 out of 545,531 sites, and 280 out of 288 accessions remained.

The tomato dataset was resequenced with a mean coverage of 36-fold, and includes accessions from 13 different species [41]. The dataset also contains raw reads and variant call files from accessions that were excluded from the original publication. These were excluded when further analysis revealed admixed ancestry (R Finkers, personal communication, February 7, 2019). We choose only to use the accessions of which the species identity was verified, and merged all single sample files using VCFTools. Due to the high coverage of this dataset, it was especially important to reduce the number of variant sites for computational efficiency. To reduce the number of variant sites to a maximum of 100,000, we briefly tested two strategies namely (1) applying a strict filter (> 80% call rate, > 1% minor allele frequency, and no indels, which kept 1.9 million out of 71.1 million variant sites) and subsequently randomly thinning to 100,000 using Plink2.0 [42], and (2) randomly thinning on all unfiltered 71.1 million variant sites using Plink2.0. We found that, for this dataset, median prediction accuracy markedly improved across classifiers (0.91 vs. 0.73) when applying the second strategy versus the first one. We therefore proceeded with this dataset using strategy 2. The resequenced tomato dataset includes 13 different species and features a major class imbalance, with 50 out of 80 accessions belonging to *Solanum lycopersicum*. Among the species with less representation are *S. corneliomulleri* and *S. galapagense*, both represented by 1 accession, and seven more species that are represented by 2 accessions. This leads to a median species size of only 2.

The distribution of species in the AFLP tomato dataset by Zuriaga et al*.* is less extreme [43]. It includes 14 different species, and 3 hybrid accessions. *S. pimpinellifolium* is represented by 26 accessions, and *S. galapagense* by 2. The other species lie somewhere in between (median = 9). The AFLP marker data were received from Zuriaga upon request. We received present/absent scoring for 245 markers in Genetix format, a format actually designed for diploid data. Thirteen of these markers had heterozygous data, which is odd because AFLPs are dominant. Zuriaga agreed these were erroneous, but retrieving the data as used for analysis 10 years ago proved difficult (personal communication, June 27, 2019). We removed all markers with heterozygous data, as well as another 13 markers with a minor allele frequency below 1%. This resulted in 219 markers for analysis.

The accession numbers of the accessions used, their a priori classifications, and the predictions of all classifiers for the resequenced tomato dataset, the sunflower dataset, and the AFLP tomato dataset can be found in Additional files 4, 5, and 6, respectively.

### Treatment of curated sunflower dataset

The number of informative SNPs present in a given genomic dataset may vary greatly depending on genotyping technique, data processing, and not in the least, crop properties. To investigate the effects of species representation and misclassification rate (and isolate them as much as possible), we selected a single expansive dataset to artificially vary species representation and misclassification rate.

Firstly, the accessions of all species with less than 10 accessions were removed. The remaining material was imported into R [44]. To avoid the confounding effect of a priori misclassifications, the dataset was screened for outliers using two very different techniques: visual inspection of a neighbour-joining tree and a Random Forest-based outlier detection method [45]. For the latter, functions *randomForest* and *outlier* from R package Random Forest were used. First *Random Forest* was run with *ntree* = 50,000 and proximity set to *TRUE* to obtain a proximity matrix of the data. This matrix describes the similarity of two individuals by counting how often they land in the same terminal node in a tree. With this matrix and the original classifications, the *outlier* function then determines which individuals have small proximities to all other cases in their class, relative to the proximities these cases have to each other.

Visual inspection of the neighbour-joining tree revealed 4 potential misclassifications accessions (Additional file 2). Using the recommended threshold of 10, the Random Forest outlier detection method flagged 6 accessions as potential outliers (Additional file 3). These accessions were all excluded from further analysis, as were the remaining *H. exilis* accessions because their group size dropped below 10. After this selection, 199 accessions from 8 species remained. From this material, 10, 8, 6, 4, and 2 samples were randomly selected from each species. These populations were used to examine model performance under varying numbers of species representation.

### Simulation of misclassifications

The goal of this part of the research is to determine which methods are most suited to correct misclassifications in genomic datasets, without the use of a gold standard dataset. To simulate these misclassifications, the species names of 6.25%, 12.5% and 18.75% of the samples were randomly altered to a random different species name from the dataset. These random alterations were introduced 5,000 times for each misclassification rate and each species representation. Each time, classifiers made predictions based on the same sets of a priori (mis)classifications. A total of 75,000 datasets were analyzed, comprising 5 different levels of species representation, 3 rates of misclassification and 5,000 replications.

### Classifier comparison on curated datasets

To quantify classifier performance, we used prediction accuracy. Prediction accuracy is a simple and intuitive metric, defined as the number of correct predictions, divided by the number of samples. Ambiguous predictions were excluded from the calculation. It must be noted however, that prediction accuracy as a summary metric must be treated with caution, as this metric is very sensitive to strong variation in the number of accessions per species. Good alternatives to prediction accuracy in imbalanced datasets are Matthews correlation coefficient (for binary predictions) and the lesser known *RK* statistic (for multiclass predictions) [46, 47]. In this case we were able to use prediction accuracy because we consistently represented all species by the same number of accessions in the curated datasets.

To test the null hypothesis that all classifiers show identical performance under all circumstances, prediction accuracies were grouped by misclassification rate and sample size, and tested using Friedman Aligned Ranks. Like the Friedman test, this is a non-parametric test that makes no assumptions about the distribution or variance of the data, and hence was considered appropriate to test the null hypothesis [48]. Friedman Aligned Ranks has been shown to perform better than the Friedman test when the number of classifiers is low, i.e. no more than 4 or 5 [49]. To correct for multiple testing, each p-value was corrected with the Finner test. This test has greater power than the conservative Bonferroni-Dunn test, and similar power to Holm, Hochberg, Hommel, Holland, and Rom, while having a simpler design [49]. If the adjusted p-value was below 0.05, it was followed up by a multiple comparison with the classifier with the highest mean accuracy as control. These statistical tests and comparisons were performed in R, using R package scmamp [50] which has been especially developed for statistical comparison of multiple algorithms.

### Classifier comparison on complete datasets

For the comparison of the complete datasets, we also used prediction accuracy. To prevent bias towards classifiers that perform well on large classes, we not only looked at the overall prediction accuracy, but also at the performance per species. Prediction accuracy per species is defined as the number of correct predictions per species, divided by total number of accessions belonging to the species. Ambiguous predictions, as are sometimes made by NJ and 3-NN, are again excluded from the calculation. The complete datasets are used without any modification, as their purpose is only to confirm whether the conclusions of the curated datasets hold, and appear generalizable. For the supervised machine learners (RF and NB) we acquired an unbiased estimate of prediction. We used the out-of-bag prediction accuracy for Random Forest as estimate, and used leave-one-out as a sampling strategy for Naive Bayes.

## Supplementary Information

**Additional file 1.** Prediction accuracy per species and species representation. Classifiers are Random Forest (RF), Naive Bayes (NB), Neighbour-Joining (NJ), 1-Nearest Neighbour (1-NN), and 3-Nearest-Neighbours (3-NN), respectively. The number of accessions per species is denoted by ’n’. The best performance for each dataset is presented in bold.

**Additional file 2.** Neighbour-Joining tree of Helianthus species represented by 10 or more accessions. The number of divergent sites was used as a measure of distance. Potentially misclassified accessions (niv07, pet02, max148, and pet88 ) are marked by a black asterisk.

**Additional file 3.** RandomForest outlier scores for all sunflower accessions. The dashed line represents the cut-off score used, which is 10.

**Additional file 4.** Tomato reseq dataset and predictions. List of resequenced tomato accessions included in this study, their a priori classifications and species predictions from all classifiers studied.

**Additional file 5.** Sunflower dataset and predictions. List of sunflower accessions included in this study, their a priori classifications and species predictions from all classifiers studied.

**Additional file 6.** Tomato AFLP dataset and predictions. List of tomato AFLP accessions included in this study, their a priori classifications and species predictions from all classifiers studied.

## Data Availability

The resequenced sunflower data used for this study are available from the Sunflower Genome Database: https://sunflowergenome.org/diversity/. The sequence reads and associated analyses of the resequenced tomato data used for this study are available in the European Nucleotide Archive (http://www.ebi.ac.uk/ena/) under accession number PRJEB5235. The AFLP marker data of tomato used for this study were received from Zuriaga. The authors have permission to redistribute this data upon request. All data generated during this study are included in this published article and its supplementary information files.

## Abbreviations

CWR: Crop wild relatives
RF: Random Forest
NB: Naive Bayes
NJ: Neighbour-Joining
1-NN: 1-Nearest Neighbour
3-NN: 3-Nearest Neighbours
LOO: Leave-one-out cross-validation
OOB: Out-of-bag
