# Supplementary material for: Reliable genomic strategies for species classification of plant genetic resources
Source: BMC Bioinformatics. 2021 Mar 31;22:173. doi: 10.1186/s12859-021-04018-6 (PMC8011391; doi:10.1186/s12859-021-04018-6)
Supplement: Supplementary file 2 — Additional file 2. Neighbour-Joining tree of Helianthus species represented by 10 or more accessions. The number of divergent sites was used as a measure of distance. Potentially misclassified accessions (niv07, pet02, max148, and pet88 ) are marked by a black asterisk. [file 12859_2021_4018_MOESM2_ESM.pdf]

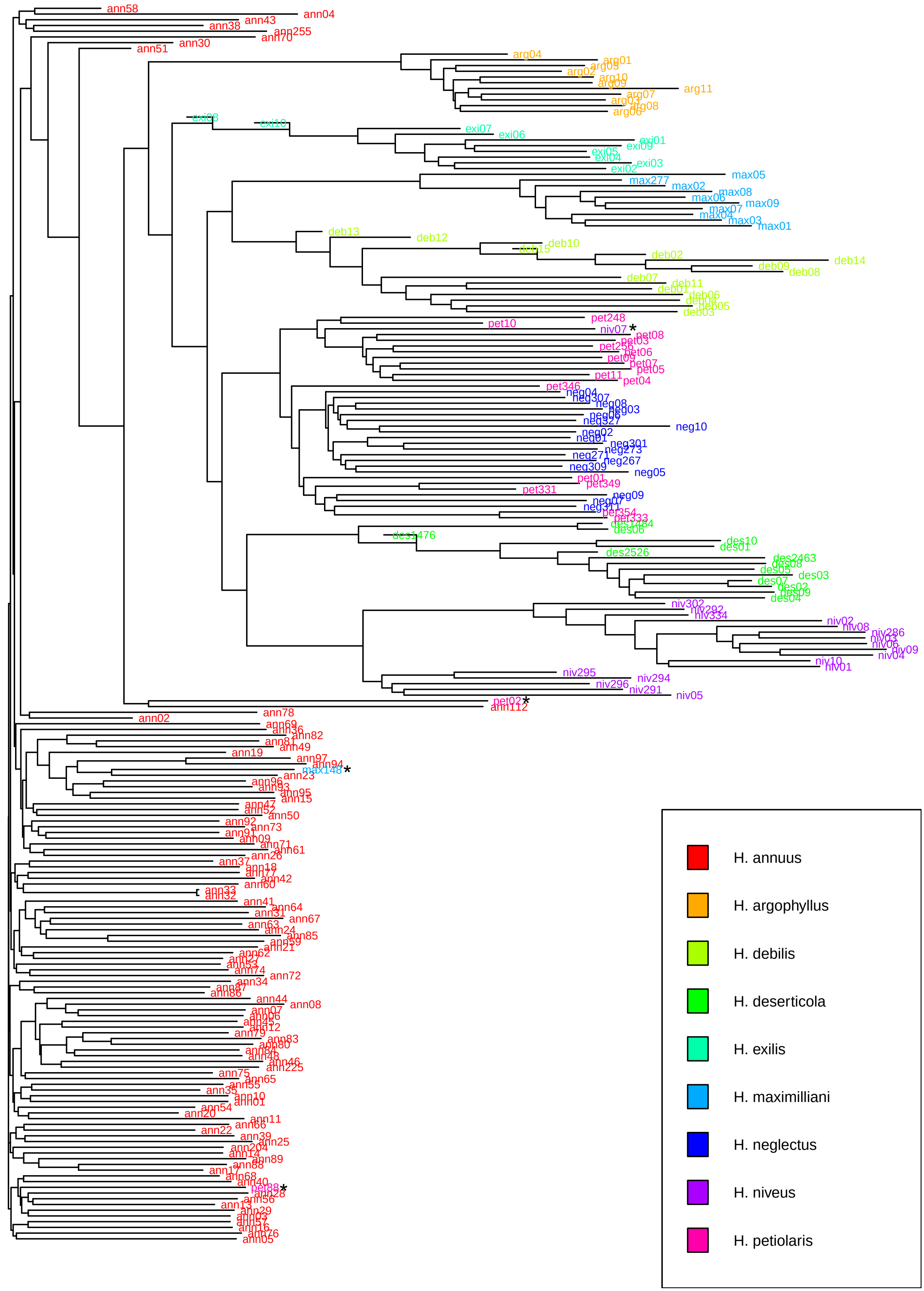

ann58
ann04
ann43
ann38
ann255
ann70
ann30
ann51
arg04
arg01
arg05
arg02
arg10
arg09
arg11
arg07
arg03
arg08
arg06
exi08
exi10
exi07
exi06
exi01
exi09
exi05
exi04
exi03
exi02
max05
max277
max02
max08
max06
max09
max07
max04
max03
max01
deb13
deb12
deb10
deb15
deb02
deb14
deb09
deb08
deb07
deb11
deb01
deb06
deb04
deb05
deb03
pet248
pet10
niv07 *
pet08
pet03
pet256
pet06
pet09
pet07
pet05
pet11
pet04
pet346
neg04
neg307
neg08
neg03
neg06
neg327
neg10
neg02
neg01
neg301
neg273
neg271
neg267
neg309
neg05
pet01
pet349
pet331
neg09
neg07
neg311
pet354
pet333
des1484
des06
des1476
des10
des01
des2526
des2463
des08
des05
des03
des07
des02
des09
des04
niv302
niv292
niv334
niv02
niv08
niv286
niv03
niv06
niv09
niv04
niv10
niv01
niv295
niv294
niv296
niv291
niv05
pet02 *
ann112
ann78
ann02
ann69
ann36
ann82
ann8
ann49
ann19
ann97
ann94
max148 *
ann23
ann96
ann93
ann95
ann15
ann47
ann52
ann50
ann92
ann73
ann91
ann09
ann71
ann61
ann37
ann26
ann18
ann77
ann42
ann60
ann33
ann32
ann41
ann64
ann31
ann67
ann63
ann24
ann85
ann59
ann21
ann62
ann27
ann53
ann74
ann72
ann34
ann87
ann86
ann44
ann08
ann07
ann06
ann45
ann112
ann79
ann83
ann80
ann84
ann48
ann46
ann225
ann75
ann65
ann35
ann55
ann10
ann01
ann54
ann20
ann11
ann66
ann22
ann39
ann25
ann204
ann14
ann89
ann17
ann88
ann68
ann40
pet88 *
ann28
ann56
ann13
ann29
ann03
ann57
ann16
ann76
ann05
H. annuus
H. argophyllus
H. debilis
H. deserticola
H. exilis
H. maximilliani
H. neglectus
H. niveus
H. petiolaris
