# Supplementary figures and images for: Reliable genomic strategies for species classification of plant genetic resources
Source: BMC Bioinformatics. 2021 Mar 31;22:173. doi: 10.1186/s12859-021-04018-6 (PMC8011391; doi:10.1186/s12859-021-04018-6)

Random Forest outlier scores for all sunflower accessions

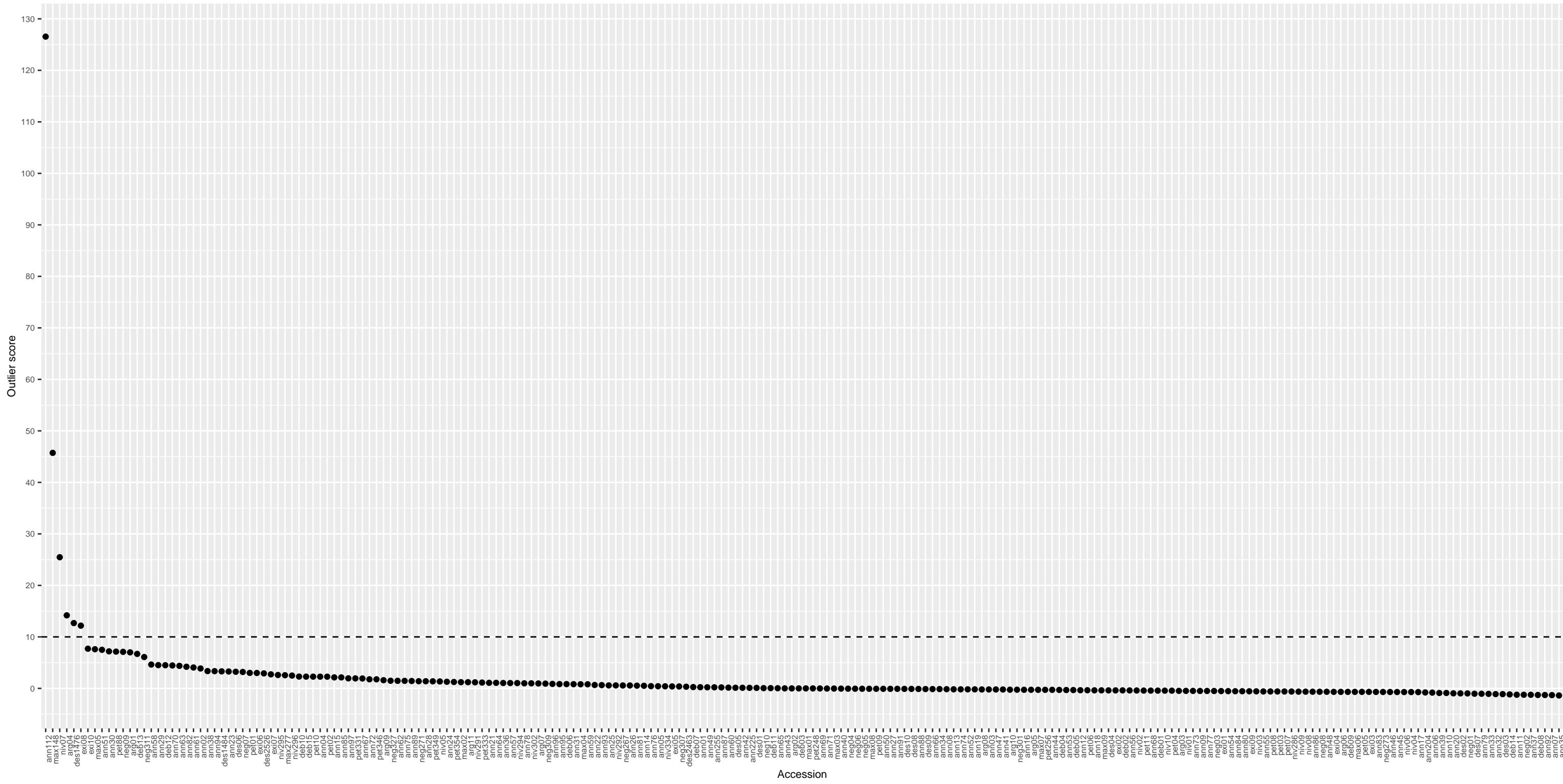

Supplement: Supplementary file 3 — Additional file 3. RandomForest outlier scores for all sunflower accessions. The dashed line represents the cut-off score used, which is 10. [file 12859_2021_4018_MOESM3_ESM.pdf]
